# Novel Function of Osteocalcin in Chondrocyte Differentiation and Endochondral Ossification Revealed on a CRISPR/Cas9 *bglap–bglap2* Deficiency Mouse Model

**DOI:** 10.3390/ijms25189945

**Published:** 2024-09-15

**Authors:** Xiang-Fang Yu, Bin Teng, Jun-Feng Li, Jian V. Zhang, Zhe Su, Pei-Gen Ren

**Affiliations:** 1 Department of Endocrinology, Shenzhen Children’s Hospital, Shenzhen 518026, China; xf.yu1@siat.ac.cn; 2 Center for Energy Metabolism and Reproduction, Shenzhen Institute of Advanced Technology, Chinese Academy of Sciences, Shenzhen 518055, China; bin.teng@siat.ac.cn (B.T.); lijf@siat.ac.cn (J.-F.L.); jian.zhang@siat.ac.cn (J.V.Z.); 3 Shenzhen College of Advanced Technology, University of Chinese Academy of Sciences, Shenzhen 518055, China; 4 Center for Cancer Immunology, Shenzhen Institute of Advanced Technology, Chinese Academy of Sciences, Shenzhen 518055, China

**Keywords:** osteocalcin, chondrocyte differentiation, endochondral ossification, cartilage

## Abstract

Endochondral ossification is the process by which cartilage is mineralized into bone, and is essential for the development of long bones. Osteocalcin (OCN), a protein abundant in bone matrix, also exhibits high expression in chondrocytes, especially hypertrophic chondrocytes, while its role in endochondral ossification remains unclear. Utilizing a new CRISPR/Cas9-mediated *bglap–bglap2* deficiency (OCN^em^) mouse model generated in our laboratory, we provide the first evidence of OCN’s regulatory function in chondrocyte differentiation and endochondral ossification. The OCN^em^ mice exhibited significant delays in primary and secondary ossification centers compared to wild-type mice, along with increased cartilage length in growth plates and hypertrophic zones during neonatal and adolescent stages. These anomalies indicated that OCN deficiency disturbed endochondral ossification during embryonic and postnatal periods. Mechanism wise, OCN deficiency was found to increase chondrocyte differentiation and postpone vascularization process. Furthermore, bone marrow mesenchymal stromal cells (BMSCs) from OCN^em^ mice demonstrated an increased capacity for chondrogenic differentiation. Transcriptional network analysis implicated that BMP and TGF-β signaling pathways were highly affected in OCN^em^ BMSCs, which is closely associated with cartilage development and maintenance. This elucidation of OCN’s function in chondrocyte differentiation and endochondral ossification contributes to a more comprehensive understanding of its impact on skeletal development and homeostasis.

## 1. Introduction

During bone developmental formation and fracture healing, endochondral ossification is an intricate and essential process whereby a cartilage template is transformed into bone [1,2]. At the onset of endochondral bone formation, the mesenchymal progenitors undergo condensations and differentiation into chondrocytes. Chondrocytes then proliferate and produce an extracellular matrix to form the primordial cartilage. Subsequently, these proliferating chondrocytes in the central region of the cartilage differentiate into hypertrophic chondrocytes and synthesize an extracellular matrix that has a different composition than that of proliferating cartilage. The hypertrophic cartilage is invaded by blood vessels, osteoblasts, osteoclasts and hematopoietic cells, resulting in the formation of primary ossification centers, a process in which the hypertrophic cartilage matrix is replaced by the novel bone matrix [3,4]. Throughout these stages, the extracellular matrix proteins exert diverse influences, including structural support, promote chondrogenesis and osteogenesis, and induce the osteoblast precursors [5].

Osteocalcin (OCN) is an abundant protein in bone matrix, and has a high expression in the chondrocytes, especially hypertrophic chondrocytes [6,7]. This availability allows OCN to always be present in the endochondral ossification microenvironment, even after series external matrix degradation or replacement occurs. Moreover, the linkage between the OCN and endochondral ossification can be supported by the clinical research. In osteoarthritis (OA), the most common cartilage disorder, OCN expression was found to increase proportionally with disease severity at both the mRNA and protein levels [8]; a rare disease, osteochondrodysplasia, suggested an association of the OCN level with progenitor cells chondrogenesis and endochondral ossification [9]. However, the conflicting functions found by other reports of OCN have interfered with its study in mineralization. In endochondral ossification, the appearance of calcium phosphate crystals signifies the onset of this process. The OCN was found to either inhibit the crystal growth by binding to Ca^2+^-rich crystal precursors via its negatively charged γ-carboxylated glutamic acid residues [10,11], or, alternatively, to promote the crystal growth by facilitating the alignment of crystals along collagen fibres [12]. Therefore, the function and mechanism of OCN in chondrocyte differentiation and endochondral ossification still remain unknown.

To investigate the role of OCN in endochondral ossification and to understand the underlying mechanisms, we established an OCN^em^ (endonuclease-mediated mutation for OCN) mouse strain utilized CRISPR/Cas9 technology. This new strain was a valuable tool to reveal the intricate functions and regulatory pathways of OCN involved in skeletal development. By comparing endochondral ossification in WT and OCN^em^ mice during early developmental stages, and by assessing the chondrogenic differentiation pathways in bone marrow mesenchymal stromal cells (BMSCs) isolated from these mice, our study sheds new light on OCN’s role as an inducer of endochondral ossification. Considering the previously obscure role of OCN in modulating cartilage, this paper offers initial insights and elucidates how OCN can suppress chondrogenic differentiation while simultaneously promoting endochondral ossification. This study may offer new avenues for future research or potential therapeutic interventions targeting OCN in conditions characterized by abnormal cartilage disease.

## 2. Results

**Figure 1:**
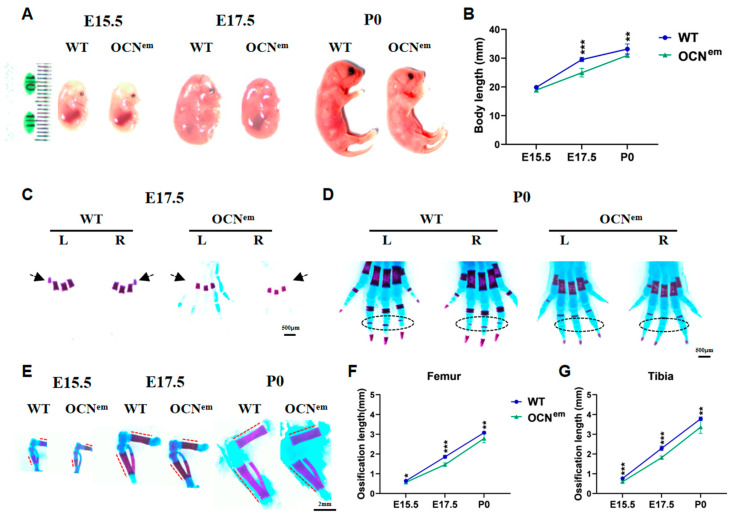
CRISPR/Cas9-mediated *bglap–bglap2* deficiency (OCN^em^) mice delayed development of early endochondral ossification and shortened primary ossification center (POC) in embryonic period. (**A**) Gross morphology of WT and OCN^em^ mice. (**B**) Body length. (**C**–**E**) Alizarin red S/alcian blue staining on hindlimbs of WT and OCN^em^ mice; L: left, R: right. (**C**) Metacarpals ossification at E17.5; arrows indicated metacarpal POC, scale bars: 500 μm. (**D**) Metacarpals/phalanges ossification at P0; ovals circled the middle phalangeal POC, scale bars: 500 μm. (**E**) Femurs and tibias ossification at E15.5, E17.5, and P0; dot lines indicated femoral and tibial POC, scale bars: 2 mm. (**F**,**G**) Quantification of the POC length of femur and tibia. There were 5 mice in each group, no samples were excluded and all data were expressed as mean ± SD. The statistical significance was denoted as * *p* < 0.05, ** *p* < 0.01, *** *p* < 0.001 by Student’s *t*-test.

By CRISPR/Cas9-mediated gene editing, we generated a new *bglap*–*bglap2* double-knockout mouse model, termed OCN^em^, which lacks OCN. Confirmed by genomic sequencing, the OCN^em^ mouse had 6743 bp deleted from the adjacent intron segment ahead of *bglap* to exon 4 of *bglap2* (Figure S1A,B); therefore, OCN^em^ mice have no full-length mRNA of *bglap* or *bglap2* expressed (Figure S1C). Long bones in mammals are formed by endochondral ossification, a process that involves the formation of the following key ossification structures: the primary ossification centres (POCs), the secondary ossification centres (SOCs) and the growth plate (GP) [13]. To elucidate the functions of OCN in early endochondral ossification, fetal WT and OCN^em^ mice were collected on embryonic day (E) 15.5 and E17.5, and postnatal day (P0). The growth retardation exophenotype was discernible at E15.5 and became evident with growth when comparing the OCN^em^ and WT mice (Figure 1A). The OCN^em^ mice have a shorter (*p* = 0.0524, *p* = 0.0003, *p* = 0.0087) body length than WT mice (Figure 1B), and delayed POCs are readily seen in the long bones of OCN^em^ mice (Figure 1C–E). At E17.5, WT mice had 4 ossified metacarpals, while there were only 3 in OCN^em^ mice (Figure 1C); at P0, a stage where the middle phalanges of WT mice had already ossified, the OCN^em^ mice had yet to show signs of ossification in these regions (Figure 1D). In addition, the POC of femurs and tibias was formed in both groups since E15.5 (Figure 1E), but the ossification length in these bones was consistently shorter (*p* = 0.0171, *p* < 0.0001, *p* = 0.0052; *p* = 0.0006, *p* < 0.0001, *p* = 0.0062) in OCN^em^ mice across all observed time points (Figure 1F,G).

**Figure 2:**
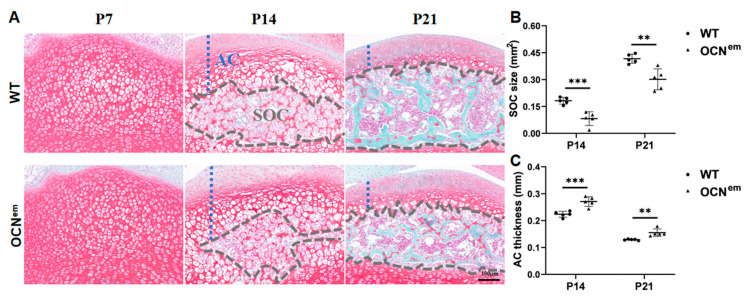
The formation of secondary ossification centers (SOCs) was delayed in OCN^em^ mice after birth. (**A**) Representative safranin O/fast green staining of the proximal tibia of WT and OCN^em^ mice at P7, P14 and P21 (Scale bars: 100 μm). AC: articular cartilage; gray dashed line outlined the SOC. Quantification of the SOC size (**B**) and AC thickness (**C**). There were 5 mice in each group, no samples were excluded and all data were expressed as mean ± SD. The statistical significance was denoted as ** *p* < 0.01, *** *p* < 0.001 by Student’s *t*-test.

The POC was generated in the diaphysis of long bones, which were delayed in the OCN^em^ embryonic period (Figure 1). Later, the endochondral ossification continues within the epiphyses, generating the SOC in the postnatal period. As expected, SOC development retardation in OCN^em^ mice was found in Figure 2A and Figure S1. From postnatal proximal tibia, the articular cartilage (AC) was unstratified, with rounded chondrocytes exhibiting a random isotropic distribution pattern at P7; from P14-P21, the SOC formed and extended. The SOC was well-developed in WT mice, but much smaller (*p* = 0.0008, *p* = 0.0039) in OCN^em^ mice (Figure 2B). Meanwhile, OCN^em^ mice showed thicker (*p* = 0.0010, *p* = 0.0031) AC than WT mice (Figure 2C). Those anomalies indicated that the deficiency of OCN disturbed endochondral ossification both in embryonic and postnatal period.

**Figure 3:**
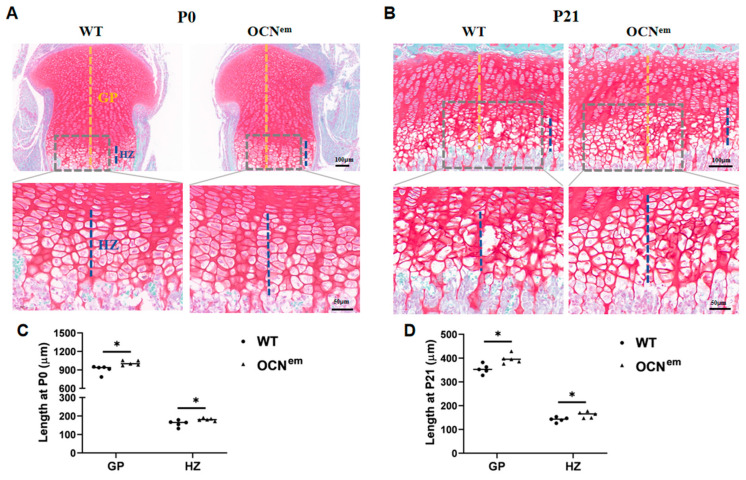
Knockout of OCN significantly increased the cartilage length. (**A**,**B**) Representative safranin O/fast green staining of tibial growth plate (GP) (upper panel) and higher magnification view of hypertrophic zone (HZ) (lower panel) of WT and OCN^em^ mice at P0 (**A**) and P21 (**B**). At upper panel, black dashed lines indicated growth plates (Scale bars: 100 μm); at lower panel, gray dashed lines outlined HZ (Scale bars: 50 μm). (**C**,**D**) Quantification of the overall length of GP, PZ and HZ at P0 (**C**) and P21 (**D**). GP: growth plate, PZ: proliferative zone, HZ: hypertrophic zone. There were 5 mice in each group, no samples were excluded and all data were expressed as mean ± SD. The statistical significance was denoted as * *p* < 0.05 by Student’s *t*-test.

Due to the endochondral ossification, the cartilage gradually declined from whole area into GP with the development of lone bone [14]. To investigate the role of OCN in endochondral ossification at the GP, we further performed the histological evaluation on the tibial cartilaginous epiphysis of WT and OCN^em^ mice at the neonatal (Figure 3A) and adolescent stages (Figure 3B). At the neonatal stage (P0), the average length of the GP in WT mice was 906.8 ± 68.6 μm, whereas in OCN^em^ mice it was 1012.5 ± 38.2 μm (Figure 3C, *p* = 0.0168). Also, the OCN^em^ mice exhibited an expanded hypertrophic zone (HZ) from 159.5 ± 17.5 μm to 181.3 ± 7.5 μm (*p* = 0.0337). As in neonatal mice, at the beginning stage of adolescence (P21), the following respective measurements show a similar trend that the GP and HZ were longer in OCN^em^ mice than in WT mice: GP length: 396.6 ± 19.7 μm, 354.2 ± 20.2 μm, HZ length: 161.9 ± 13.0 μm, 141.9 ± 10.1 μm (Figure 3D, *p* = 0.0100, *p* = 0.0260). These results reflect that there is a delay of endochondral ossification in OCN^em^ mice, which may relate to the increase in chondrocyte differentiation and cartilage formation.

**Figure 4:**
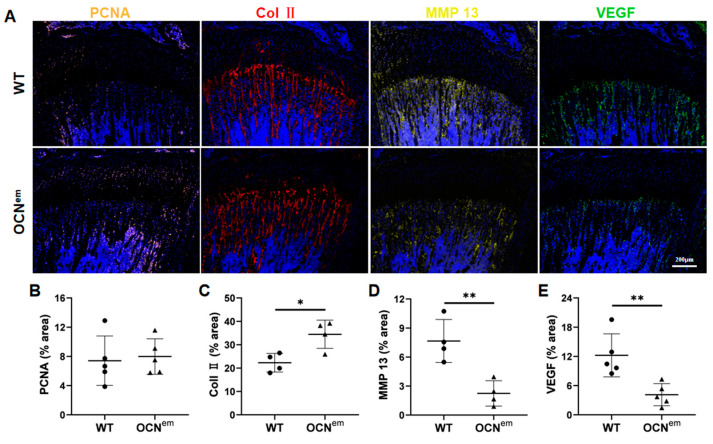
Knockout of OCN increased chondrocyte differentiation and postponed vascularization process. (**A**) IF staining to detect proliferation, differentiation and vascular invasion markers in mice growth plate. Scale bars: 200 μm; (**B**–**E**) Quantification of the expression of PCNA, Col II, MMP 13, and VEGF in the growth plate. There were 5 mice in each group, no samples were excluded and all data were expressed as mean ± SD. The statistical significance was denoted as * *p* < 0.05, ** *p* < 0.01 by Student’s *t*-test.

To elucidate the underlying mechanism of increased cartilage in OCN^em^ mice, we select several factors to conduct immunofluorescence (IF) staining including proliferation, differentiation, ECM degradation and vasal invasion of tibial cartilage tissue (Figure 4A and Figure S1). The pattern of proliferation marker, proliferating cell nuclear antigen (PCNA), was comparable between OCN^em^ and WT mice (Figure 4B), indicating that the cartilage expansion was not a result of chondrocyte proliferation. Collagen (Col) II, a collagen type specific to chondrocytes, serves as a definitive marker of chondrocyte differentiation. Its expression was significantly elevated (*p* = 0.0151) in OCN^em^ mice compared to WT controls (Figure 4C), suggesting an enhancement in chondrocyte differentiation in the absence of OCN. Matrix metalloproteinase (MMP) 13 is instrumental in the degradation of extracellular matrix proteins within cartilage, thereby facilitating vascular invasion—a key step in endochondral ossification. Notably, the expression of MMP 13 was markedly reduced (*p* = 0.0057) in the growth plates of OCN^em^ mice (Figure 4D). Additionally, vascular endothelial growth factor (VEGF), a crucial mediator of angiogenesis, exhibited a downward trend (*p* = 0.0067) (Figure 4E), signifying a diminished vascular invasion in the growth plate of OCN^em^ mice. Collectively, these findings suggest that the increase cartilage in OCN^em^ mice may be a consequence of increasing chondrocytes differentiation and postponing vascularization process, which imply a delay in the endochondral ossification process within the growth plate.

**Figure 5:**
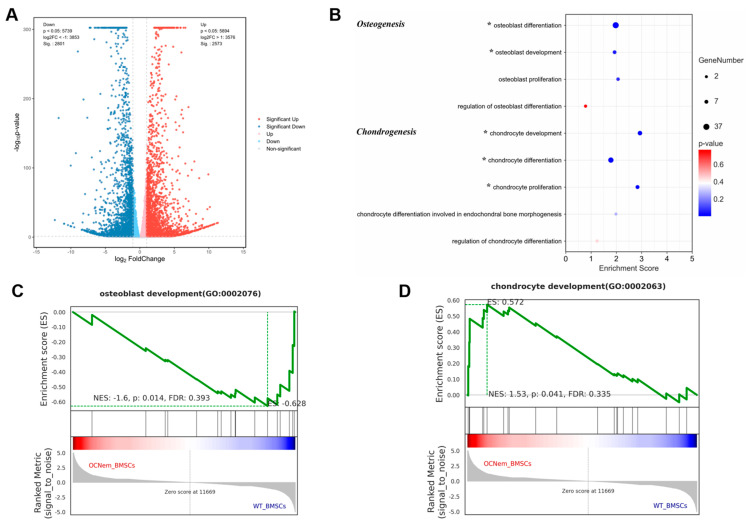
Knockout of OCN decreased the differentiation potential of bone marrow mesenchymal stromal cells (BMSCs) to bone, but more importantly increased the differentiation potential to cartilage. (**A**) A volcano plot illustrating differentially regulated gene expression between the WT and OCN^em^ BMSCs. Values are presented as the log10 of tag counts; (**B**) The GO functional clustering of differentially expressed genes associated with chondrogenesis and osteogenesis process. * means this biological process was affected; (**C**,**D**) GSEA analyzed the genes associated with osteoblast development and chondrocyte development. NES, normalized enrichment score; FDR, false discovery rate. There were 3 samples in each group and no samples were excluded.

To explore the function and mechanism of OCN in chondrocyte differentiation, we carried out related experiments in vitro. Due to osteoblasts and chondrocytes being both derived from a common mesenchymal progenitor [15], BMSCs, we isolated them from newborn WT and OCN^em^ mice (P0). Then, BMSC RNA-sequencing was performed to gain more insight into the role of OCN in differentiation. In reference to WT, 2801 down-regulated genes and 2573 up-regulated genes were found in the OCN^em^ BMSCs (*p*-value < 0.05 & |log2FC| > 1) (Figure 5A). In further cluster analysis of differential expression genes (DEGs), as illustrated in Figure S1, *bglap* and *bglap2* were found to be absent in OCN^em^ BMSCs. Concurrently, a notable up-regulation was observed in genes pivotal for chondrogenesis, including *Col2a1*, *Comp* and *Cnmd,* among others (Figure S1). The subsequent gene ontology (GO) enrichment analysis results indicated that several biological processes were significantly affected especially around the osteogenesis and chondrogenesis (Figure 5B). Gene Set Enrichment Analysis (GSEA) analysis also suggested that OCN deficiency reduced the differentiation potential of BMSCs to bone, while increased the differentiation potential to cartilage (Figure 5C,D).

**Figure 6:**
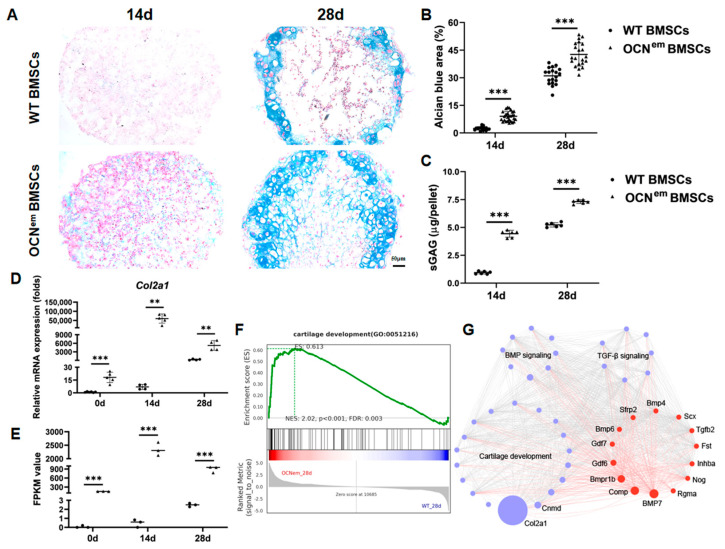
Chondrogenic differentiation of BMSCs from OCN^em^ mice was significantly enhanced. (**A**) Photomicrographs of WT BMSCs and OCN^em^ BMSCs pellets on days 14 and 28 stained with alcian blue/nuclear fast red staining. Scale bars: 50 μm; (**B**) Quantified the percentage of alcian blue positive area; (**C**) sGAG content in WT BMSCs and OCN^em^ BMSC pellets on day 14 and 28; (**D**,**E**) Detection of chondrogenic differentiation–related genes *Col2a1* expression by RT–qPCR and RNA–sequencing; (**F**) GSEA analyzed the genes associated with cartilage development; (**G**) Transcriptional network of the differentially expressed genes related to cartilage development, BMP signaling and TGF–β signaling. The node size was set based on foldchange in the differentially expressed genes. Red-colored circles indicate genes present in 2 or more purple–colored circles. There were more than 3 samples in each group, no samples were excluded and all data were expressed as mean ± SD. The statistical significance was denoted as ** *p* < 0.01, *** *p* < 0.001 by Student’s *t*-test.

To investigate the effect of OCN in chondrogenic differentiation, BMSCs were incubated for micromass culture in chondrogenic differentiation medium. From the morphology of pellet, there were significant differences between the WT and OCN^em^ BMSC groups. The pellets from OCN^em^ BMSCs could be easily clipped and transferred, while pellets of WT BMSCs were easily crushed with tweezers, which might indicate that the stiffness and agglomeration of the pellets were different. Moreover, alcian blue/nuclear fast red staining suggested that there were more (*p* < 0.0001, *p* < 0.0001) chondrocytes differentiated from OCN^em^ BMSCs (Figure 6A,B). Quantifying sulfated glycosaminoglycan (sGAG) content per pellet showed that OCN^em^ BMSCs pellets had much more (*p* < 0.0001, *p* < 0.0001) proteoglycan secretion at 14 or 28 days (Figure 6C). Real-time quantitative reverse transcription PCR (RT–qPCR) analysis in chondro-pellets also exhibited enhanced (*p* < 0.0001, *p* = 0.0021, *p* = 0.0011) expression of chondrogenesis markers such as *Col2a1* (Figure 6D), *Sox9*, *Acan*, etc. (Figure S1) in OCN^em^ BMSCs. In our transcriptome data, we also observed a significant upregulation (*p* < 0.0001, *p* < 0.0001, *p* = 0.0001) of *Col2a1* (Figure 6E), which further confirmed our qPCR results. After 28 days of differentiation, the OCN^em^ BMSC RNA-sequencing revealed significantly up-regulated genes related to cartilage development (Figure 6F). Subsequently, we examined the upstream regulatory pathways of chondrogenesis, BMP and TGF-β, which are two key pathways closely involved in the regulation of cartilage formation and development. Transcriptional network analysis showed that the genes differentially expressed during cartilage development strongly interacted with the BMP and TGF-β signaling pathways (Figure 6G). Among these, BMP7 emerges as a central hub linking the two signaling pathways, demonstrating gene upregulation in differentiated osteocalcin-deficient BMSCs pellets and protein increase in OCN^em^ mice (Figure S1).

## 3. Discussion

The present study introduces a novel perspective on the role of OCN in endochondral ossification, utilizing the CRISPR/Cas9-engineered OCN^em^ mouse model. Our findings demonstrated the intricate relationship between OCN and the regulation of chondrocyte differentiation and endochondral ossification. The delay in endochondral ossification observed in OCN^em^ mice is associated with increased chondrocyte differentiation and the formation of cartilage, highlighting the potential of OCN as an inducer of endochondral ossification.

Endochondral ossification is a crucial process in skeletal development, leading to the formation of the POC in the diaphysis (central part of bone) during the embryonic period, the SOC at the ends of the bone (epiphysis) and a narrow growth plate (epiphyseal plate) between the POC and SOC in long bones during adolescence [16]. It plays a dual role in replacing embryonic cartilage during organ development and extending long bones to maturity. Despite its importance, there is limited research on the effects of specific proteins or mechanisms in endochondral ossification across different stages and anatomical sites. In our study, we explored the role of OCN in the differentiation of chondrocytes and the endochondral ossification, encompassing the development of the POC, SOC and growth plate throughout embryonic, neonatal, and adolescent phases. Our research findings, as depicted in Figure 1 and Figure 2, suggest that mice with a mutation in the OCN gene (OCN^em^) exhibit a retardation in the development of both the POC and SOC. In the neonatal phase, the epiphyses are entirely cartilaginous, with the primary growth plate beginning to take shape between the cartilaginous epiphysis and the POC. As adolescence progresses, the SOCs gradually form into roughly spherical structures within the cartilage, while the growth plate concurrently narrows [17,18]. The absence of OCN is associated with an elongation of the growth plate during the early stages of life, as illustrated in Figure 3.

Our investigation has revealed that the lack of OCN results in an increased differentiation of chondrocytes and a postponed vascularization process, as shown in Figure 4, which impedes the advancement of endochondral ossification. By isolating BMSCs from WT and OCN^em^ mice, we have uncovered a significant enhancement in the chondrogenic potential of BMSCs in the absence of OCN by RNA-sequencing and bioinformatics analyses, as depicted in Figure 5. The deficiency in OCN seems to alter the developmental trajectory of BMSCs, biasing their differentiation towards a chondrogenic fate rather than an osteogenic one. This redirection of the BMSCs’ lineage commitment could have profound implications for our understanding of skeletal development.

The commitment of BMSCs to specific cell fates is a multifaceted journey governed by an intricate tapestry of signaling pathways, including Wnt, TGF-β, BMP and FGF [19,20]. Dysregulation of these pathways have been implicated in numerous hereditary skeletal diseases in humans [21,22,23]. Notably, the BMP and TGF-β signaling pathways, which are crucial for the regulation of cartilage development and maintenance. These pathways are prominently featured in our study, as depicted in Figure 6. Transcriptional network analysis points towards a heightened interaction between these pathways and the differentially expressed genes during cartilage development in OCN^em^ BMSCs. Notably, BMP7 emerged as a pivotal signaling molecule within these pathways and is highly expressed in OCN^em^ mice. We hypothesize that the absence of OCN may establish a protective effect on cartilage via a BMP7-mediated mechanism, thereby delaying subsequent stages of endochondral ossification. This hypothesis is supported by evidence that BMP7 can promote the synthesis of essential cartilage matrix components and enhance the production of proteoglycans and collagen [24,25]. Moreover, BMP7 has protective and repair effects on cartilage, can reduce the degradation of articular cartilage in OA [26] and can delay the progression of cartilage degeneration [27]. Therefore, we further believe that the delayed endochondral ossification observed in OCN^em^ mice attributed to increased chondrocyte differentiation and cartilage formation may serve as a potential regulatory and drug target for osteoarthritis through the BMP7 pathway.

While our study provides compelling evidence for the role of OCN in endochondral ossification, it also acknowledges the limitations that necessitate further research. The dynamics of the OCN–BMP7 interaction in skeletal development require a more comprehensive elucidation. Additionally, the potential role of OCN in osteoarthritis, given the established protective effects of BMP7 on cartilage [26,28], warrants investigation. However, due to limitations in research scale and time, we did not assess the differentiation ability of OCN^em^ BMSCs to hypertrophic chondrocytes in vitro or evaluate the function of OCN on OA model in vivo. Future studies will aim to bridge these gaps, expanding our understanding of OCN’s multifaceted roles in skeletal development and homeostasis.

In summary, our research presents a working model for chondrocytes differentiation and endochondral ossification. By leveraging the OCN^em^ mouse model, we have uncovered OCN’s potential as a regulatory factor that influences the balance between chondrocyte differentiation and skeletal formation. These findings not only enhance our molecular understanding of skeletal development but also hold promise for the development of therapeutic strategies targeting OCN in conditions associated with aberrant bone and cartilage development.

## 4. Materials and Methods

### 4.1. Generation of OCN^em^ Mice and Housing

The C57BL/6J mice were purchased from GemPharmatech (Nanjing, China). The OCN^em^ mice were generated by CRISPR/Cas9 gene-editing; CRISPR single-guide RNAs were designed to intron ahead of *bglap* (ATTACCTCGTCGGGGAGT) and exon 4 of *bglap2* (GGAGCAGTGTGAGCTTAAC) in the OCN locus. The mutant mice genotype was identified using the following primers: WT-F (CCAAATCCCCTTGGCTTCTGA) and WT-R (TCTAGCCCTCTGCAGGTCATAGAG) to amplify a 225 bp wild type (WT) product. In a separate reaction, KO-F (TGAGGACATTACTGACCACTCCCTC) and KO-R (CCCCTATTACCACCACAATGGAC) were used to amplify a 687 bp mutant product. The WT and OCN^em^ mice were maintained under a 12 h light–dark cycle with *ad libitum* access to food and water at the Shenzhen Institute of Advanced Technology (SIAT), Chinese Academy of Sciences Animal Facility. The animal care procedures followed the guidelines of the Ethics Committee for Animal Research, protocol number: SIAT-IACUC-200106-YYS-RPG-A1005.

### 4.2. Whole-Mount Skeletal Staining

Fetal mice were subjected to whole-mount alizarin red S/alcian blue staining [29]. Briefly, mice (5–7 mice per group) were eviscerated and fixed in 95% ethanol for 7 days and transferred into acetone for another 7 days at room temperature; they were then stained in 5 vol 0.1% alizarin red S (A5533, Merck, Darmstadt, Germany) in 95% ethanol, 1.5 vol Alcian–Blue staining solution (TMS-010, Merck, Germany), 5 vol 100% acetic acid and 88.5 vol 70% ethanol at 37 °C for 3 days. After rinsing with ddH_2_O, specimens were transferred into 0.5% KOH (*w*/*v*) at 4 °C until the muscle tissue was transparent and then transferred into 1% KOH at room temperature until the skeletons became visible after approximately 48 h. After the staining, cartilage and mineralized bone appear blue and red, respectively. For long-term storage, specimens can be soaked from 20%, 50%, and finally to 80% glycerol solution containing 1% KOH (*w*/*v*). Images of hindlimbs were taken with a stereoscopic microscope (SMZ745, Nikon, Tokyo, Japan), and the ossification lengths of the femur and tibia were quantified by Image J software 1.46r [30].

### 4.3. Histological and Immunofluorescence (IF) Staining

Tibias (5 mice per group) were fixed with paraformaldehyde (G1101, Servicebio, Wuhan, China), decalcified in EDTA (G1105, Servicebio, China), paraffin embedded (39601095, Leica, Wetzlar, Germany), 5 μm sectioned and stained with safranin O/fast green staining kit (G1371, Solarbio, Beijing, China). Briefly, the deparaffinized and rehydrated sections were stained in Weigert’s iron hematoxylin followed by 0.02% aqueous fast green and rinsing in 1% acetic acid and 0.1% aqueous safranin O. The sections were imaged using a microscope (BX53, Olympus, Tokyo, Japan). Stained sections were used for morphometric measurements of articular cartilage, secondary ossification center, hypertrophic zone and growth plate. The start of the hypertrophic zone was defined as the point at which the disc shape cells of the proliferative zone started to round up and become larger; the end of the zone was taken as the vascular invasion front [31]. The growth plate heights were measured in the central part of the growth plate section by measuring heights of the resting, proliferative and hypertrophic zones along the longitudinal axis.

For IF staining [32], tibias were fixed, decalcified, embedded and sectioned as above. Primary antibodies specific to PCNA, Col II, MMP 13, VEGF (GB12010, GB11021, GB11247, GB15165, Servicebio, Wuhan, China) and BMP7 (sc-53917, Santa Cruz, Dallas, TX, USA) were applied at dilutions of 1:500, 1:2000, 1:2000, 1:3000 and 1:1000, respectively. Following incubation with the primary antibodies, fluorochrome-conjugated second-antibody were used for detection. After DAPI (G1012, Servicebio, Wuhan, China) counterstaining, images were obtained under a fluorescent microscope (Nikon Eclipse C1, Nikon, Japan). The mean areas of interest regions were measured on immunostained images by Image J software 1.46r.

### 4.4. Isolation, Cultivation and Chondrogenic Differentiation of BMSCs

After euthanizing the newborn mice (3 mice per group), the bilateral hindlimbs were isolated, metaphysis removed and medulla flushed out. The cell suspension was filtered through a 70 mm filter mesh to remove any bone spicules, muscle and cell clumps. A single-cell suspension was prepared in ice-cooled PBS and centrifuged at 1000 rpm for 5 min, supernatant discarded and cells resuspended in MEM Alpha Modification medium (10% FBS and 1% P/S). Suspended primary cells were plated in a 25 cm^2^ culture bottle and cultured in 37 °C and 5% CO_2_ incubator. The medium was changed every 3 days and cell growth and morphology were observed under an inverted microscope (IX73P1F, Olympus, Japan). Cells were passaged at 1:3 after lifting by 0.25% trypsin when they almost merged [33].

For chondrogenic differentiation [34], BMSCs were cultured at high-density micromass (pellets of 3 × 10^5^ cells) in complete MesenCult™–ACF Chondrogenic Differentiation Medium (#05455, Stemcell, Vancouver, Canada). Formed pellets were cultured in 15 mL polypropylene tubes for 28 days, keeping the cap on with a half twist.

### 4.5. RNA-Sequencing and Bioinformatics Analyses

The total RNA of BMSCs and chondrocyte differentiated (3 samples per group) were trizol extracted (1559608, ThermoFisher, Waltham, MA, USA) and RNA integrity was assessed by Agilent 2100 Bioanalyzer (Agilent Technologies, Santa Clara, CA, USA). Then, libraries were constructed using VAHTS Universal V6 RNA-sequencing Library Prep Kit according to the manufacturer’s instructions. The libraries were sequenced on an Ilumina Novaseq 6000 platform and 150 bp paired-end reads were generated. Raw reads of fastq format were first processed using fastp and the low-quality reads were removed to obtain the clean reads. The clean reads were mapped to the reference genome using HISAT2. The FPKM of each gene was calculated and the read counts of each gene were obtained by HTSeq-count. The transcriptome sequencing and analysis were conducted by OE Biotech Co., Ltd. (Shanghai, China).

Differential expression analysis was performed using the DESeq2. The Q value < 0.05 and foldchange > 2 or foldchange < 0.5 were set as the threshold for significantly DEGs. The GO enrichment analysis of DEGs was performed to screen the significantly enriched term using R (v3.2.0). The GSEA was performed using GSEA software v2.0.1.8. The interaction for genes related to cartilage, BMP signaling, and TGF-β signaling was searched using the STRING database and further analyzed using Cytoscape based on the degree of connectivity of the nodes [35].

### 4.6. Alcian Blue/Nuclear Fast Red Staining of Chondrogenic Pellet

After chondrogenic differentiation, pellets were fixed in 4% paraformaldehyde (AR1068, Boater, Wuhan, China) for 30 min. Then, the pellets were embedded in an optimum cutting temperature compound (4583, SAKURA, Torrance, CA, USA) and frozen at −20 °C. Embedded frozen tissues were sectioned in 10 μm thickness using a cryostat (CM1950, Leica, Germany). For alcian blue/nuclear fast red staining [36], the sections were covered with Alcian–Blue staining solution (TMS-010, Merck, Germany) for 30 min. Then, the slides (4 samples per group, 4 sections per sample) were rinsed with running water until the excess alcian blue was removed from the slides. The sections were counter-stained using nuclear fast red (100121, Merck, Germany) for 10 min, washed with running water, and dehydrated with graded ethanol. The sections were imaged using a microscope (BX53, Olympus, Japan).

### 4.7. Sulfated Glycosaminoglycan (sGAG) Quantification

The total sGAG quantifications were carried out after pellet (6 samples per group) digestion with papain extraction reagent (P3125, Sigma, Livonia, MI, USA) at 65 °C for 3 h with occasional mixing. The synthesized sGAG in the pellets was measured in triplicate by Blyscan sGAG assay (B110, Biocolor, Carrickfergus, Italy), chondroitin 4-sulfate used as a reference standard. The optical density (OD) was measured at 656 nm wavelength (Multiskan GO, ThermoFisher, Waltham, MA, USA).

### 4.8. Real-Time Quantitative Reverse Transcription PCR (RT–qPCR)

Reverse transcription of Trizol extracted mRNA was performed using a reverse transcription PCR kit (RR036, Takara, Japan). The RT–qPCRs were carried out using a light cycler (Roche, Basel, Switzerland) and SYBR quantitative real-time PCR kit (RR820, Takara, Osaka, Japan). The expression of genes associated with chondrogenic differentiation was amplified and detected using specific primers sets, the sequences of which are detailed in Figure S1. For the quantitative analysis of BMP7 mRNA levels, a Mouse Bmp7 qPCR Primer Pair (QM01390S, Beyotime, Shanghai, China) was utilized. The qPCR data were analyzed using ∆Ct method, with gene expression normalized to the endogenous control of the GAPDH gene.

### 4.9. Statistical Analysis

All data were presented as the mean ± standard deviation. The *p* values were analyzed by Student’s *t*-test on GraphPad Prism version 8.0.0 for Windows, San Diego, CA, USA. They were marked as *: *p* < 0.05, **: *p* < 0.01, ***: *p* < 0.001, respectively.

## Data Availability

The original contributions presented in the study are included in the article, further inquiries can be directed to the corresponding authors. The raw sequencing results can be accessed with the accession number PRJNA1152645 in the NCBI’s Sequence Read Archive.

## Supplementary Materials

The supporting information can be downloaded at: https://www.mdpi.com/article/10.3390/ijms25189945/s1.

## References

[1] Wuelling M., Vortkamp A. (2011). Chondrocyte proliferation and differentiation. Endocr. Dev..

[2] Mackie E.J., Ahmed Y.A. (2008). Endochondral ossification: How cartilage is converted into bone in the developing skeleton. Int. J. Biochem. Cell Biol..

[3] Sun M.M., Beier F. (2014). Chondrocyte hypertrophy in skeletal development, growth, and disease. Birth Defects Res. C Embryo Today.

[4] Aghajanian P., Mohan S. (2018). The art of building bone: Emerging role of chondrocyte-to-osteoblast transdifferentiation in endochondral ossification. Bone Res..

[5] Grzelkowska-Kowalczyk K. (2016). The importance of extracellular matrix in skeletal muscle development and function. Composition and Function of the Extracellular Matrix in the Human Body.

[6] Bernabei I., So A. (2023). Cartilage calcification in osteoarthritis: Mechanisms and clinical relevance. Nat. Rev. Rheumatol..

[7] Grissom S.K., Semevolos S.A. (2023). Role of cartilage and bone matrix regulation in early equine osteochondrosis. Bone Rep..

[8] Pullig O., Weseloh G. (2000). Chondrocyte differentiation in human osteoarthritis: Expression of osteocalcin in normal and osteoarthritic cartilage and bone. Calcif. Tissue Int..

[9] Wyckoff M.H., El-Turk C. (2005). Neonatal lethal osteochondrodysplasia with low serum levels of alkaline phosphatase and osteocalcin. J. Clin. Endocrinol. Metab..

[10] Romberg R.W., Werness P.G. (1986). Inhibition of hydroxyapatite crystal growth by bone-specific and other calcium-binding proteins. Biochemistry.

[11] Ducy P., Desbois C. (1996). Increased bone formation in osteocalcin-deficient mice. Nature.

[12] Moriishi T., Ozasa R. (2020). Osteocalcin is necessary for the alignment of apatite crystallites, but not glucose metabolism, testosterone synthesis, or muscle mass. PLoS Genet..

[13] Moncayo-Donoso M., Guevara J.M. (2019). Morphological changes of physeal cartilage and secondary ossification centres in the developing femur of the house mouse (*Mus musculus*): A micro-CT based study. Anat. Histol. Embryol..

[14] Setiawati R., Rahardjo P. (2019). Bone development and growth. Osteogenesis and Bone Regeneration.

[15] Hu L., Yin C. (2018). Mesenchymal Stem Cells: Cell Fate Decision to Osteoblast or Adipocyte and Application in Osteoporosis Treatment. Int. J. Mol. Sci..

[16] Mackie E.J., Tatarczuch L. (2011). The skeleton: A multi-functional complex organ: The growth plate chondrocyte and endochondral ossification. J. Endocrinol..

[17] White A., Wallis G. (2001). Endochondral ossification: A delicate balance between growth and mineralisation. Curr. Biol..

[18] Wilson K., Usami Y. (2021). Analysis of Association between Morphometric Parameters of Growth Plate and Bone Growth of Tibia in Mice and Humans. Cartilage.

[19] Chen G., Deng C. (2012). TGF-beta and BMP signaling in osteoblast differentiation and bone formation. Int. J. Biol. Sci..

[20] Chen Q., Shou P. (2016). Fate decision of mesenchymal stem cells: Adipocytes or osteoblasts?. Cell Death Differ..

[21] Joyce M.E., Roberts A.B. (1990). Transforming growth factor-beta and the initiation of chondrogenesis and osteogenesis in the rat femur. J. Cell Biol..

[22] Kawakami Y., Rodriguez-Leon J. (2006). The role of TGFbetas and Sox9 during limb chondrogenesis. Curr. Opin. Cell Biol..

[23] Wu M., Wu S. (2024). The roles and regulatory mechanisms of TGF-beta and BMP signaling in bone and cartilage development, homeostasis and disease. Cell Res..

[24] Chubinskaya S., Kuettner K.E. (2003). Regulation of osteogenic proteins by chondrocytes. Int. J. Biochem. Cell Biol..

[25] Flechtenmacher J., Huch K. (1996). Recombinant human osteogenic protein 1 is a potent stimulator of the synthesis of cartilage proteoglycans and collagens by human articular chondrocytes. Arthritis Rheum..

[26] Badlani N., Oshima Y. (2009). Use of bone morphogenic protein-7 as a treatment for osteoarthritis. Clin. Orthop. Relat. Res..

[27] Hayashi M., Muneta T. (2010). Intra-articular injections of bone morphogenetic protein-7 retard progression of existing cartilage degeneration. J. Orthop. Res..

[28] Hurtig M., Chubinskaya S. (2009). BMP-7 protects against progression of cartilage degeneration after impact injury. J. Orthop. Res..

[29] Tang C.Y., Wu M. (2021). Runx1 is a central regulator of osteogenesis for bone homeostasis by orchestrating BMP and WNT signaling pathways. PLoS Genet..

[30] Schneider C.A., Rasband W.S. (2012). NIH Image to ImageJ: 25 years of image analysis. Nat. Methods.

[31] Kung L.H., Rajpar M.H. (2015). Increased classical endoplasmic reticulum stress is sufficient to reduce chondrocyte proliferation rate in the growth plate and decrease bone growth. PLoS ONE.

[32] Stickens D., Behonick D.J. (2004). Altered endochondral bone development in matrix metalloproteinase 13-deficient mice. Development.

[33] Maridas D.E., Rendina-Ruedy E. (2018). Isolation, Culture, and Differentiation of Bone Marrow Stromal Cells and Osteoclast Progenitors from Mice. J. Vis. Exp..

[34] Xu J., Li D. (2020). Comparison of skeletal and soft tissue pericytes identifies CXCR4(+) bone forming mural cells in human tissues. Bone Res..

[35] Kim P., Park J. (2022). Mast4 determines the cell fate of MSCs for bone and cartilage development. Nat. Commun..

[36] Antunes J.C., Tsaryk R. (2015). Poly(gamma-Glutamic Acid) as an Exogenous Promoter of Chondrogenic Differentiation of Human Mesenchymal Stem/Stromal Cells. Tissue Eng. Part. A.

