# Supplementary material for: Novel Function of Osteocalcin in Chondrocyte Differentiation and Endochondral Ossification Revealed on a CRISPR/Cas9 bglap–bglap2 Deficiency Mouse Model
Source: Int J Mol Sci. 2024 Sep 15;25(18):9945. doi: 10.3390/ijms25189945 (PMC11431882; doi:10.3390/ijms25189945)
Supplement: Supplementary file 1 [file ijms-25-09945-s001.zip › ijms-3180785-supplementary.pdf]

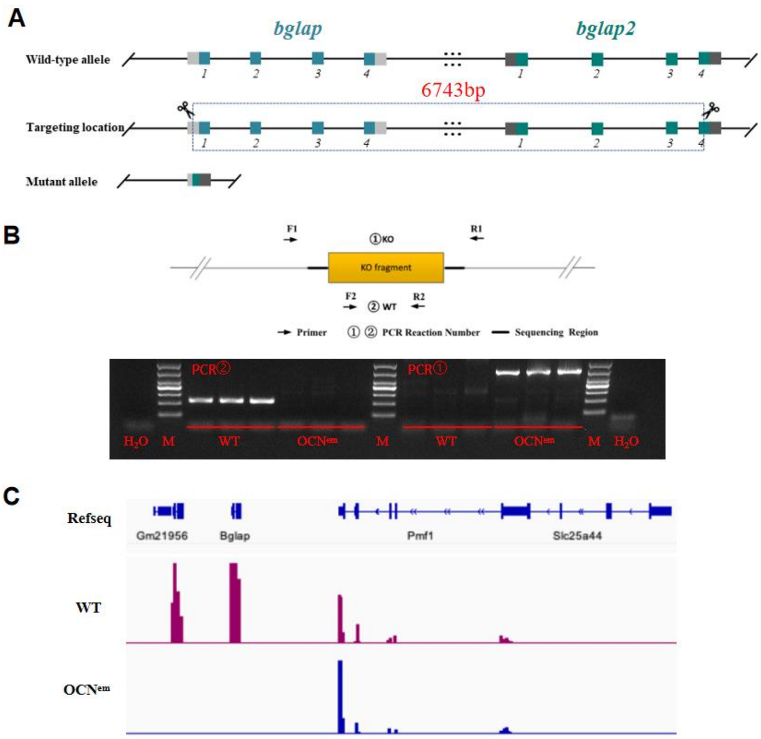

Figure S1. Successful establishment of OCN^{em} mice. (**A**) A schematic representation of strategy for editing *bglap* and *bglap2* gene by CRISPR/Cas9 technology; (**B**) Primers designed for OCN^{em} mice gene identification and agarose gel electrophoresis identified of the mice tail DNA from WT and OCN^{em} mice in littermates. H_2O was the negative control; M: DNA marker; (**C**) The absence of sequencing reads were mapping to *Bglap* and *Bglap2* exons in OCN^{em} mice.
